# Development and In Vitro Evaluation of Oral Capsules from *Antiaris*: A Convenient Substitute for Peripheral Neuropathy

**DOI:** 10.1155/2022/5340953

**Published:** 2022-04-27

**Authors:** Mary-Ann Archer, Doris Kumadoh, Samuel Nii-Bortier Gaizer, Adelaide Mensah, Jonathan Jato, Micheal Odoi Kyene, Susana Oteng Mintah, Genevieve Naana Yeboah, Paul kwesi Sodzi, Ofosua Adi-Dako

**Affiliations:** ^1^Department of Pharmaceutics, University of Cape Coast, Cape Coast, Ghana; ^2^Department of Drug Production and Quality Assurance, West African Postgraduate College of Pharmacist, Accra, Ghana; ^3^Department of Pharmaceutics and Quality Control, Centre for Plant Medicine Research, Mampong-Akuapem, Ghana; ^4^Department of Production, Centre for Plant Medicine Research, Mampong-Akuapem, Ghana; ^5^Department of Pharmaceutics, School of Pharmacy, University of Health and Allied Sciences, Ho, Ghana; ^6^Department of Pharmacognosy and Herbal Medicine, School of Pharmacy, University of Health and Allied Sciences, Ho, Ghana; ^7^Department of Microbiology, Centre for Plant Medicine Research, Mampong-Akuapem, Ghana; ^8^Department of Pharmaceutical Sciences, Sunyani Technical University, Sunyani, Ghana; ^9^Department of Pharmaceutics and Microbiology, School of Pharmacy, University of Ghana, Legon, Accra, Ghana

## Abstract

*Antiaris* is a monoherbal decoction produced by the Centre for Plant Medicine Research (CPMR), Mampong-Akuapem, Ghana. It is prepared from the stem bark of *Antiaris africana* Engl. (Moraceae), prescribed, and dispensed to patients for the management of nervous disorders. This current formulation presents notable challenges in patients' adherence to treatment regimen due to its bulkiness and bitterness. These challenges have resulted in a decrease in therapeutic outcome. This study sought to transform *Antiaris* into oral capsules to mask its bitter taste and reduce bulkiness of the product to improve patients' convenience. In this study, four (4) conventional release capsule formulations were successfully prepared from the decoction via wet granulation using corn starch, lactose, light magnesium carbonate (LMC), and microcrystalline cellulose (MCC) and labelled A01, A02, A03, and A04 respectively. The drug-excipient compatibility studies on A01, A02, A03, and A04 were investigated using Fourier transform infrared (FTIR) spectroscopy. The flow properties of the granules as well as the quality assessment of the formulations such as dissolution, disintegration, uniformity of weight, and assay tests were evaluated using pharmacopoeial and nonpharmacopoeial methods. Appropriate models were used to investigate the difference factor (*f*_*1*_) and similarity factor (*f*_*2*_) of the dissolution profiles of the formulations and *Antiaris*. From the study, all formulated granules had excellent flow properties with Carr's index from 7.83 to 9.56%, Hausner's ratio from 1.09 to 1.10, and angle of repose from 25.13 to 27.87°. Drug-excipient compatibility studies demonstrated no interaction between extract and used excipients. All formulations passed the uniformity of weight, disintegration, assay, and dissolution tests. Formulation A02 had the highest dissolution efficiency of 100.12%, while A03 recorded the least value of 97.22% in the 1 h dissolution studies. A comparison of their various dissolution profiles, respectively, to that of its decoction demonstrated their similarity, since, in all comparisons, *f*_2_ < 15 and *f*_1_ > 50. This implies that, any of these four formulations could be a good substitute for *Antiaris*.

## 1. Introduction

Peripheral neuropathy is a nervous disorder where there is damage to the peripheral nerves, usually characterized by pain, numbness, burning sensation, tingling, and muscle weakness. Peripheral neuropathy is among the top ten reported cases of the outpatient clinic of CPMR (https://www.cpmr.org.gh, accessed on 18/11/20 at 3 : 00 pm). It is commonly associated with diabetes and has been reported to affect 50% of diabetic patients [1]. Other causes include alcohol abuse, chemotherapy, HIV, leprosy, infections, exposure to toxins, ageing, and injury [2].

The use of plants for treatment of ailments is the earliest known healthcare system to man [3]. The general perception that herbal medicines are safer, readily available, and are more affordable than orthodox medicines has led to a global increase in their consumption [4–6]. This high demand has necessitated the formulation and reformulation of herbal medicines into suitable dosage forms to enhance patient's convenience and compliance to achieve better therapeutic outcomes. Solid dosage forms are mostly preferred over liquid formulations because of increased stability, ability to mask bitter taste, easier to handle and carry, improved patient's compliance, and the use of less excipients.

*Antiaris* is a monoherbal decoction prepared from the stem bark of *Antiaris toxicaria* subsp. *africana* (Engl.) cc. Berg [7], family Moraceae. It is produced and marketed in Ghana by the Centre for Plant Medicine Research (CPMR), Mampong-Akuapem, for the treatment of nervous disorders. CPMR has used this herbal preparation to treat peripheral neuropathy for over two decades. Studies on this decoction have demonstrated that it possesses significant antineuropathic property at therapeutic doses which are safe [8, 9]. However, these studies also reported that the formulation in its current form as a decoction causes significant challenges in patient's compliance to treatment regimen due to its bitter taste and bulkiness. These factors may result in decreased therapeutic outcomes. Hence, this study aimed at reformulating *Antiaris* into capsules to mask the bitter taste and reduce its bulkiness to make it convenient for patients to carry everywhere they go. This will also help to reduce batch-to-batch variation so as to obtain a standardized product.

## 2. Materials and Methods

### 2.1. Materials

The starch, lactose, microcrystalline cellulose, and light magnesium carbonate were obtained from Unichem Industry Ghana Limited, who purchased them from Shanghai Shenma Pharmaceutical Technology Company Limited, Shanghai, China. Talc was obtained from the Chemical store, Department of Pharmaceutics, Kwame Nkrumah University of Science and Technology (KNUST).

### 2.2. Phytochemical Screening

Preliminary screening for the presence of secondary metabolites in the aqueous extract of the stem bark of *Antiaris africana* was identified according to methods described by Trease and Evans [10].

### 2.3. Plant Collection and Authentication

The stem bark of *Antiaris africana* was collected from CPMR's Arboretum, Mampong-Akuapem, Ghana (5^o^55′ 06.6^″^ N, 0° 07′57′57 W), by the Plant Development Department at CPMR. Voucher specimen (CPMR 5067) has been placed at CPMR's herbarium. Plant identification was achieved via comparison of the collected voucher specimens to already identified specimen at CPMR herbarium. The nomenclature and classification of the species of plants follows the Plant List database (https://www.theplantlist.org; accessed on 10/11/2020).

### 2.4. Processing and Preparation of *Antiaris* Decoction

The stem bark was sorted to remove all foreign matter. It was chopped into smaller pieces, washed under running tap water, air dried, and then milled into coarse powder. The decoction was prepared according to classified preparatory method by the staff of the Production Department of CPMR without the addition of preservatives. The decoction was then stored in a refrigerator at 4°C and used when needed for analysis.

### 2.5. Determination of UV Maximum Wavelength of Absorption of Aqueous Extract of *A. africana*

The maximum wavelength of absorption (*λ*max) of the extract was determined by scanning various concentrations (0.001091–0.1091% w/v) in distilled water using the UV-Vis spectrophotometer (Merck industries, Germany) through a wavelength range of 200–500 nm using the quartz cuvettes over a path length of 1 cm [11].

### 2.6. Formulation and Evaluation of *Antiaris* Granules for Encapsulation

**Figure 1:**
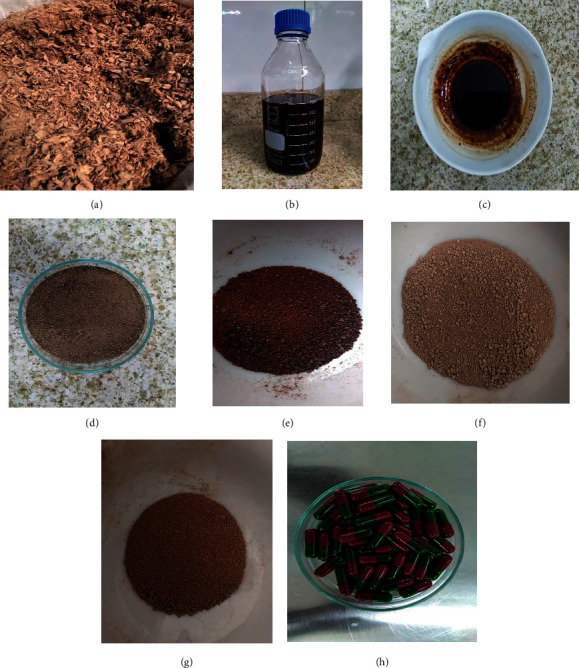
Milled stem bark of *Antiaris africana* (a). Prepared decoction/aqueous extract (b). Concentrated extract (c). A01 granules (d). Granules for A02 (e). Granules for A03 (f), Granules for A04 (g). Encapsulated granules (h).

Oral conventional release capsules were prepared using 157 mg of the aqueous extract of the stem bark of *A. africana* with same concentration of different absorbents (starch, lactose, microcrystalline cellulose (MCC), and light magnesium carbonate (LMC)), as given in Table 1. A volume of the aqueous extract equivalent to half the dose of the decoction was concentrated by evaporating in an hot air oven at 60°C. The concentrate (Figure 1) was mixed with the absorbents to form a uniform extract-excipient mixture which was further dried. The dried granular mass of different dried mixtures was screened through a sieve with mesh size 1.18 mm to produce granules with uniform size as shown in Figure 1. The flow properties of the *Antiaris* granules for encapsulation were assessed using reported methods [12, 13]; the tapped and bulk densities were used to determine Hausner's ratio and Carr's index, whereas the fixed height method was used to determine the angle of repose. The granules of the different formulations from the aqueous extract were lubricated and filled into hard gelation capsule shells (capsule size 0) using an encapsulation machine (GMP Industries, India).

### 2.7. Extract-Excipient Compatibility Studies

This study was conducted on the extract and the granules using the PerkinElmer Fourier transform infrared spectrophotometer (spectrum 2, SR. No. 94133, UK). The extract was placed on a diamond crystal, and pressure was applied with the use of the force gauge to ensure maximum contact with the test sample. It was then scanned 24 times to generate a spectrum at 4000–400/cm. This was repeated for A01, A02, A03, and A04 granules [14].

### 2.8. Evaluation of *Antiaris* Capsules

#### 2.8.1. Uniformity of Weight

Twenty capsules were randomly selected from formulation A01 and weighed altogether (SN: AE 436647 Adam Equipment, UK). One capsule from A01 was weighed and recorded. The capsule was opened, and the contents were removed as completely as possible. The emptied shell was also weighed. The net weight per capsule was determined by subtracting the weight of shell from the weight of the intact capsule. This procedure is repeated for the rest of the 19 capsules. The average net weight was determined from the sum of the individual net weights. The percentage deviation from the average net weight of each capsule was determined. The above was repeated for formulations A02, A03, and A04 [11].

#### 2.8.2. Capsule Disintegration Time Test

Six capsules from formulation A01 were randomly selected, and one capsule was dropped into each of the cylindrical glass tubes of the disintegrating apparatus (Type: ZT3/1, Erweka® GmbH, Heusenstamm, Germany). The baskets containing the tubes with the capsules (a disc was placed on each to prevent it from floating) were lowered into the beaker containing distilled water at 37 ± 2°C. The up and down movement of the basket was started, and the time taken for the last capsule to disintegrate and pass through the mesh was recorded for each capsule. The average time for the disintegration of the six capsules was calculated as its disintegration. This was repeated for formulations A02, A03, and A04 [11].

#### 2.8.3. Analysis of Drug Content of Capsules

Ten (10) capsules were randomly selected from formulation A01. Each capsule was emptied and crushed. The active ingredient was extracted with distilled water in 100 mL volumetric flask. The amount of *Antiaris africana* Engl. extract in each capsule was determined using a UV spectrophotometer at wavelength of 292 nm. This was done in triplicate, and the same procedure was repeated for A02, A03, and A04 formulations [15].

#### 2.8.4. In Vitro Extract Release Studies

This study was carried out using the USP dissolution apparatus 1 in 900 mL of distilled water at a speed of 100 rpm and temperature of 37 ± 0.5°C. Three capsules from each formulation were introduced into the consecutive round bottom beakers at 5 minutes intervals and the procedure conducted under sink conditions. At 0, 5, 10, 15, 30, 45, and 60 min, 10 mL of each sample was withdrawn and replaced with fresh dissolution medium maintained at 37 ± 0.5°C. The samples withdrawn were then filtered through a Whatman filter paper (No. 5) and assayed using the UV spectrophotometer at wavelength of 292 nm using the appropriate regression data obtained from the calibration plots of plant extract (*y* = 4.9413*x* + 0.003; *R*^2^ = 0.9865). The cumulative drug release was calculated and plotted against time [16].

### 2.9. Difference and Similarity Factors

The difference (*f1*) and similarity factors (*f2*) for the dissolution profile of the decoction (aqueous extract) as compared to the release of the extract from formulations A01, A02, A03, and A04 were determined using the model independent approach.

(1) $$\begin{array}{c} \text{Difference factor }\left(f1\right)=\frac{\sum \left(Rt-Tt\right)}{\sum \left(Rt\right)}\times 100,\\ \text{Similarity factor}\left(f2\right)=50\;\log\left[{\left[1+\left(\frac{1}{n}\times \sum {\left(Rt-Tt\right)}^{2}\;\right)\right]}^{-0.5\;}\times 100\right], \end{array}$$

where *n* is the time points, Rt is the cumulative percentage dissolved at time *t* for the reference, and Tt is the cumulative percentage dissolved at time *t* for the test [17]

### 2.10. Analysis of Data

Results from this study are presented in mean ± standard deviation. The data were statistically analysed using both Microsoft excel and GraphPad Prism, version 6 (GraphPad Software Inc., San Diego, CA, USA).

## 3. Results and Discussion

### 3.1. Phytochemical Composition of *Antiaris*

The preliminary phytochemical screening of the aqueous extract showed the presence of reducing sugar, phenolic compounds, polyuronides, saponins, triterpenes, and phytosterols. Moronkola and her team also reported the presence of tannins, flavonoids, phenols, alkaloids, reducing sugar, terpenoids steroids, anthraquinones, and cardiac glycosides in the methanolic extract of the stem bark of this plant [18]. These secondary metabolites present in the extract could be responsible for its antinociceptive property.

### 3.2. Spectrum for UV Maximum Wavelength of Absorption

**Figure 2:**
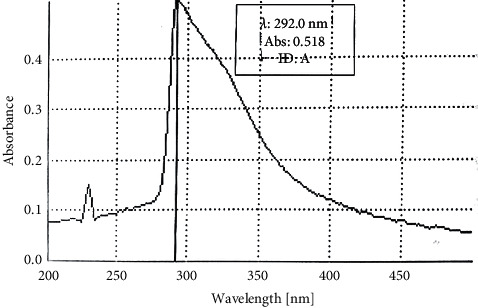
UV spectrum of aqueous extract of *Antiaris africana* (A).

The *Antiaris* decoction at concentration of 0.1091 mg/mL demonstrated two major peaks appearing at 230 nm and 292 nm (*λ*max), as shown in Figure 2. The presence of the characteristic peak at 230 nm was also reported in a similar study by [19], which is an indication of the presence of carboxyl groups of organic acid constituents of the plant [20].

### 3.3. Extract-Excipient Interaction

**Figure 3:**
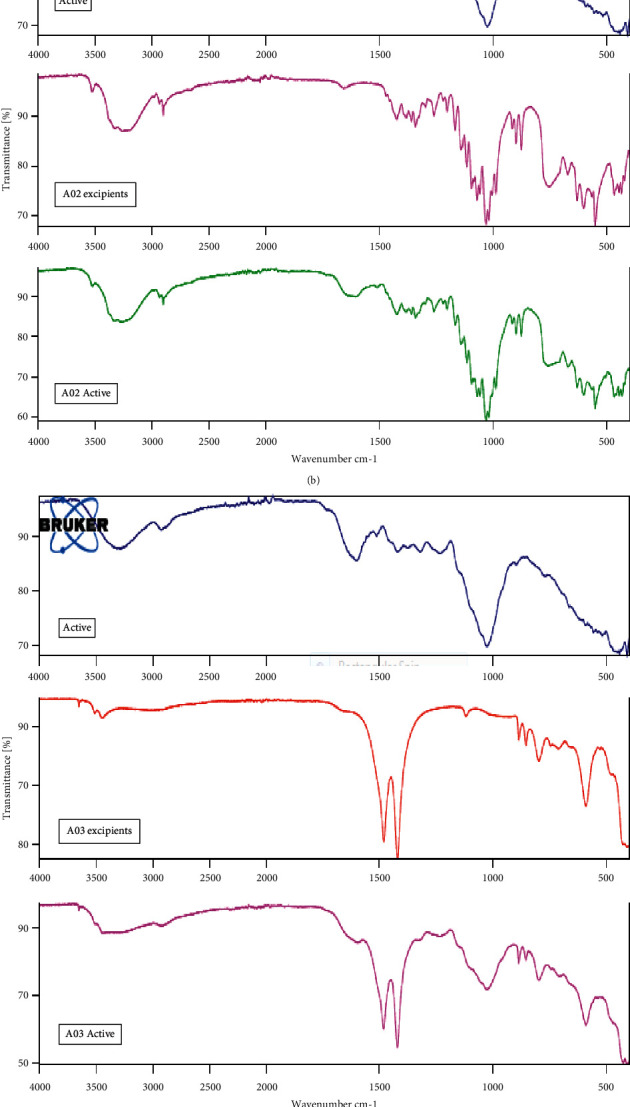
(a) FT-IR spectra of the aqueous extract of the stem bark of *Antiaris africana* (active), excipients used in formulation A01 (A01 excipients), and the mixture of extract and excipients used in A01 formulation (A01 active) showing no extract-excipient interaction. (b) FT-IR spectra of the aqueous extract of the stem bark of *Antiaris africana* (active), excipients used in formulation A02 (A02 excipients), and the mixture of extract and excipients used in A02 formulation (A02 Active) showing no extract-excipient interaction. (c) FT-IR spectra of the aqueous extract of the stem bark of *Antiaris africana* (active), excipients used in formulation A03 (A03 excipients), and the mixture of extract and excipients used in A03 formulation (A03 active) showing no extract-excipient interaction. (d) FT-IR spectra of the aqueous extract of the stem bark of *Antiaris africana* (active), excipients used in formulation A04 (A04 excipients), and the mixture of extract and excipients used in A04 formulation (A04 Active) showing no extract-excipient interaction.

There were no significant extract-excipients interactions in the FTIR, as shown in Figures 3(a)–3(d) for formulations A01, A02, A03, and A04, respectively. This is because, there was no disappearance of characteristic functional peaks of the extract in the spectra. Thus, the spectrum for the extract showed a broad weak hydroxyl band at 3276 cm^−1^, which slopes into the aliphatic region of 3000 cm^−1^, a peak between 1597.9 cm^−1^, which is a weak to medium band, which represents a C=C bond stretch of cyclic alkenes. There is also a strong peak at 1023 cm^−1^, representing a C-N bond stretch of amines. All these characteristic peaks were all present in the spectra for formulations A01, A02, A03, and A04. This result means that, starch, lactose, light magnesium carbonate, microcrystalline cellulose, and talc are inert to the extract; thus, they are ideal pharmaceutical excipients.

### 3.4. Flow Properties of *Antiaris africana* Granules

Physical evaluation of the prepared granules showed excellent flow properties according to Hausner's ratio, angle of repose, and Carr's index (Table 2). Several studies conducted on reformulation of decoctions into capsules have demonstrated that the use of adsorbents ensure effortless processing of the extracts for granules formulation and encapsulation [21–23]. This excellent flow demonstrates their suitability to be encapsulated as a result of uniform filling of capsule shell during the process of encapsulation [12].

### 3.5. Quality Evaluation of *Antiaris africana* Capsules

#### 3.5.1. Uniformity of Weight of *Antiaris africana* Capsules

The average weight for all the formulations were more than 300 mg; hence, with twenty randomly selected capsules per formulation used for this study, not more than two (2) capsules should deviate from the mean weight by more than ± 7.5% and none should deviate by ± 15% [24]. From the results (Table 3), all the formulated capsules of *Antiaris africana* passed the uniformity of the weight test. This could be as a result of the good flow properties of the granules, the even particle size distribution of the granules, even filling of the capsule shell, and even compression of the granules after filling the shell. Therefore, it is anticipated that these formulated capsules would contain a uniform dose of the extract between individual capsules due to the uniform distribution of extract in the formulations.

Standard deviation, which is a measure of the variability around the mean weight of the twenty randomly selected capsules from each formulation, demonstrated that formulation A02 had the best uniformity of weight variation due to its least standard deviation of ±0.001, whereas A01 with the highest standard deviation value of ±0.006 demonstrated a high dispersion of capsule weight from the mean weight. This makes the weight of capsules of formulation A01 the least uniform.

#### 3.5.2. Capsule Disintegration Time

The process of disintegration is an important step for drug release from immediate release dosage forms. This test is the first approach of a drug becoming absorbable. This is because this study gives data on the probable bioavailability of the drug in the body unaccompanied by in vivo studies. Formulations which fail the disintegration test might not dispense the active ingredient promptly to ensure the achievement of the desired therapeutic effect. Also, long disintegration time can lead to the disintegration of capsules in unsuitable part of the gastrointestinal tract such as the colon and rectum [25]. All the formulations disintegrated in less than 30 minutes (Table 4) at 37 ± 2°C in distilled water and hence passed the disintegration test [11]. This means that A01, A02, A03, and A04 would release the extract within the desired period for dissolution to take place.

#### 3.5.3. Assay of *Antiaris africana* Capsules

The assay of any pharmaceutical formulation is a very important quality control measure. This helps not only in determining the presence and content of active ingredients in a formulation but also aids in the elimination of counterfeit and substandard drugs from the market. A batch has a high tendency to have uniform dose per individual capsule if there are similarities in the size, density, and shape of active ingredients and excipients; the particles of the blend should have no electrostatic charge, and the mixing time should just be adequate (short mixing time could result in improper mixing of ingredients, and long mixing time could also result in overmixing which causes separation of ingredients depending on their individual properties) [26].

All the formulated *Antiaris africana* capsules had their drug content within the BP stipulated range, thus 85–115% [11], as given in Table 5. This is an indication that A01, A02, A03, and A04 may exert the needed therapeutic response and may eliminate any unexpected side effects as a result of overdosing or underdosing of extract.

#### 3.5.4. In Vitro Dissolution Studies of *Antiaris africana* Capsules

**Figure 4:**
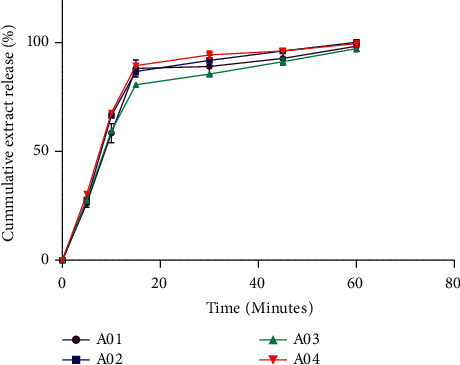
In vitro release profile of *Antiaris africana* extract from formulated capsules (mean ± *S*, *n* = 6).

This study is the backbone of quality assessment tools in evaluating the suitability of a formulation in any drug development process. It is an in vitro bioequivalent test to determine the dissolution profile as well as to compare these profiles. This aids in establishing similarities between pharmaceutical dosage forms containing the same active ingredient and also to differentiate between the effects of manufacturing variables on the release of active ingredient [27–29]. The results obtained (Figure 4) show that all the capsules passed the dissolution test since more than 70% of the extract was released from each formulation within 45 minutes as specified in the British Pharmacopoeia for all conventional immediate release capsules [11]. It was also observed that, for all formulations, more than 85% of the labelled extract was dissolved within 30 min. This means that formulations A01, A02, A03, and A04 are rapidly dissolving drug products [24]. Thus, A01, A02, A03, and A04 are suitable for patient's consumption since they would rapidly dissolve in the physiological solution of the body to dispense the extract for the occurrence of pharmacological activity.

#### 3.5.5. Comparison of Dissolution Efficiency

**Figure 5:**
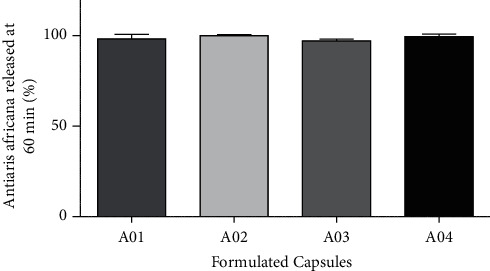
Percentage release of *Antiaris africana* extract from A01, A02, A03, and A04 capsules (mean ± SD, *n* = 3).

This study only characterizes the release of a drug, but it is not a parameter for comparative dissolution kinetics. It gives information on the consistency in each formulation. The dissolution efficiency study is suitable for quantitative comparison among formulated capsules. The higher the dissolution efficiency, the more efficient the formulation is at releasing embedded drug [30]. The result obtained (Figure 5) shows that A03 recorded the least dissolution efficiency of 97.22%, whereas A02 recorded the highest dissolution efficiency of 100.12%. It can be inferred that formulation A03 was the least efficient in the release of *Antiaris africana* extract, while A02 was the most efficient in releasing the embedded extract. However, this release differences are not significant (*p* < 0.05) to imply that one formulation may be superior to the other.

### 3.6. Assessment of Similarity (*f*2) and Difference (*f*1) Factors between *Antiaris* Decoction and Formulated Capsules

Comparison of the dissolution curves of two products (the reference and test) containing the same active ingredient is done to ensure similarity (*f2*) or difference (*f1*) in product performance to assess the possibility of a substitute. The dissolution profiles of the test and the reference drugs are identical if *f2* equals 100. However, they are said to be similar if *f2* value is between the values 50 and 100 [31, 32]. Normally, *f1* values up to 15 demonstrates minor difference between the two products [33]. The smaller the *f1,* the greater the cumulative amount of test drug dissolved across all the time points. The dissolution profiles of two products are regarded to be bioequivalent and similar if *f1* is between 0 and 15, while *f2* is also between the values 50 and 100 [34]. From the results (Table 6), A04 gave the lowest *f1*value of 3.54 and the highest *f2* value of 72.93. This can be inferred that among the four (4) formulated capsules, A04 is the most similar to the marketed decoction. However, all the *Antiaris africana* formulations were similar and bioequivalent to the decoction served on the market; hence, they can be used as alternatives for the decoction.

## 4. Conclusion

Oral conventional release capsules have been successfully developed from *Antiaris* decoction using starch, lactose, microcrystalline cellulose, and light magnesium carbonate as diluents. These formulations passed all the nonpharmacopoeial and pharmacopoeial tests. The developed capsules would help reduce batch-to-batch variation, mask the bitter taste, and reduce the bulkiness of the decoction. Hence, could be used in place of the decoction for the treatment of neuropathy to enhance patients' compliance and reduced therapy failure from bulky oral liquid formulations.

## Figures and Tables

**Table 1:** Composition of *Antiaris* capsules.

Ingredients (mg)	Formulation code			
	A01	A02	A03	A04
*A. africana* stem bark extract	157	157	157	157
Starch	338	—	—	—
Lactose	—	338	—	—
LMC	—	—	338	—
MCC	—	—	—	338
Talc	5	5	5	5
Total weight per capsule	500	500	500	500

**Table 2:** Flow properties of *Antiaris africana* granules (*n* = 3).

Formulation code	Bulk density (mg/mL)	Tapped density (mg/mL)	Carr's index (%)	Hausner's ratio	Angle of repose (°)
A01	0.79 ± 0.037	0.86 ± 0.044	7.90 ± 0.370	1.08 ± 0.006	27.87 ± 1.449
A02	0.82 ± 0.019	0.91 ± 0.00	9.56 ± 2.113	1.10 ± 0.023	26.81 ± 0.757
A03	0.52 ± 0.008	0.57 ± 0.019	7.83 ± 2.627	1.09 ± 0.031	24.58 ± 0.716
A04	0.45 ± 0.00	0.50 ± 0.025	9.10 ± 4.540	1.10 ± 0.055	25.13 ± 0.888

**Table 3:** Uniformity of weight of *Antiaris africana* capsules (*n* = 20).

Formulation code	Total net weight (g)	Average net weight (g)	No. of capsules deviating by ± 5%	No. of capsules deviating by ± 10%	Inference
A01	10.083	0.504 ± 0.006	Nil	Nil	Passed
A02	10.200	0.510 ± 0.001	Nil	Nil	Passed
A03	9.916	0.496 ± 0.004	Nil	Nil	Passed
A04	9.990	0.500 ± 0.003	Nil	Nil	Passed

Nil means no capsule deviated.

**Table 4:** Disintegration time of *Antiaris africana* capsules (*n* = 6).

Formulation code	Average disintegration time (min)
A01	6.50 ± 0.495
A02	3.58 ± 0.028
A03	6.60 ± 0.099
A04	3.25 ± 0.057

**Table 5:** Comparative percentage content of *Antiaris africana* extract in capsules (*n* = 3).

Formulation code	Average absorbance	Average drug content (%)
A01	0.440 ± 0.008	101.39 ± 0.099
A02	0.445 ± 0.059	102.55 ± 0.042
A03	0.427 ± 0.071	98.38 ± 0.015
A04	0.430 ± 0.035	99.07 ± 0.021

**Table 6:** Similarity factor (*f2*) and difference factor (*f1*) between formulated capsules and *Antiaris* decoction.

Formulation code	Similarity factor (*f2*)	Difference factor (*f1*)	Inference
A01	8.59	55.15	Similar
A02	5.32	66.69	Similar
A03	10.86	50.97	Similar
A04	3.54	72.93	Similar

## Data Availability

The data used to support the findings of this study are available from the corresponding author upon request.
